# Supplementary material for: Genome organization in double-stranded DNA viruses observed by cryoET
Source: bioRxiv. 2023 Dec 16:2023.12.15.571939. Preprint. [Version 1] doi: 10.1101/2023.12.15.571939 (PMC10760162; doi:10.1101/2023.12.15.571939)
Supplement: Supplement 13 [file NIHPP2023.12.15.571939v1-supplement-13.pdf]

bioRxiv preprint doi: https://doi.org/10.1101/2023.12.15.571939; this version posted December 16, 2023. The copyright holder for this preprint (which was not certified by peer review) is the author/funder, who has granted bioRxiv a license to display the preprint in perpetuity. It is made available under aCC-BY 4.0 International license.

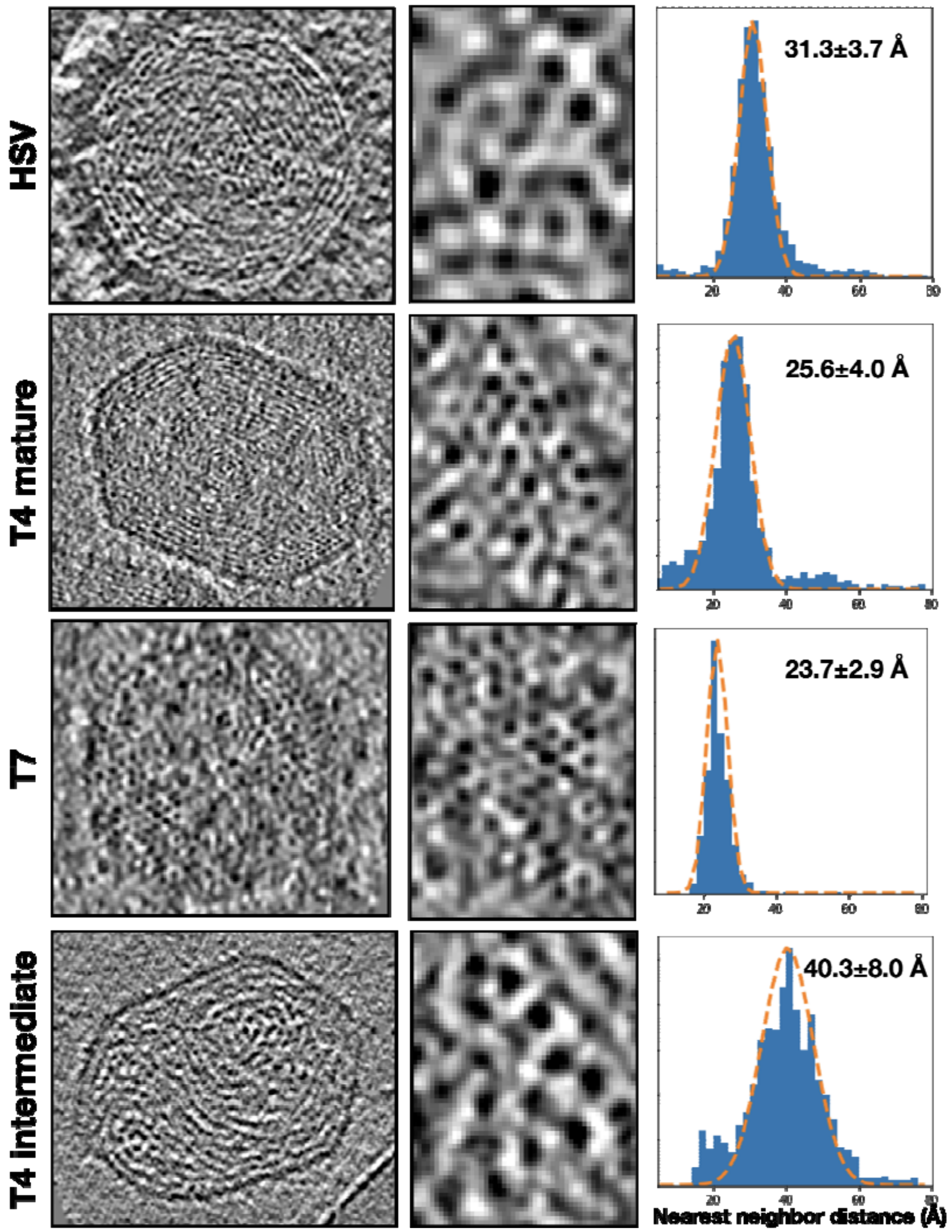


**Fig.S1.** Distribution of the distance between the nearest dsDNA strains in different viruses and different assembly states. Left column: slice view of polished subtomograms. Middle column: zoomed-in view of DNA bundle. Right column: Histogram of nearest neighbor distance distribution, with fitted Gaussian distribution overlaid.

bioRxiv preprint doi: https://doi.org/10.1101/2023.12.15.571939; this version posted December 16, 2023. The copyright holder for this preprint (which was not certified by peer review) is the author/funder, who has granted bioRxiv a license to display the preprint in perpetuity. It is made available under aCC-BY 4.0 International license.

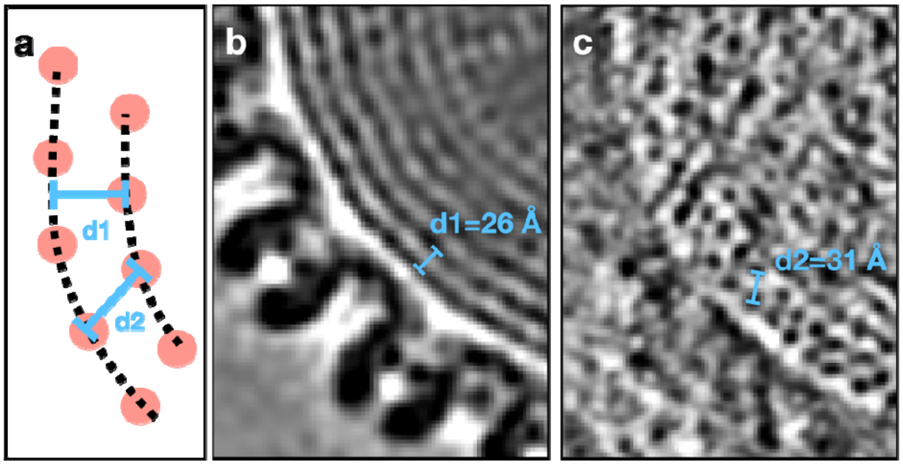


**Fig.S2.** Difference between measurement of DNA spacing from the averaged structure and individual particles. (a) Diagram showing the difference between the two measurements. (b) Measurement of DNA spacing from the averaged structure of HSV-1. (c) Measurement of DNA spacing from one polished subtomogram of HSV-1.

bioRxiv preprint doi: https://doi.org/10.1101/2023.12.15.571939; this version posted December 16, 2023. The copyright holder for this preprint (which was not certified by peer review) is the author/funder, who has granted bioRxiv a license to display the preprint in perpetuity. It is made available under aCC-BY 4.0 International license.

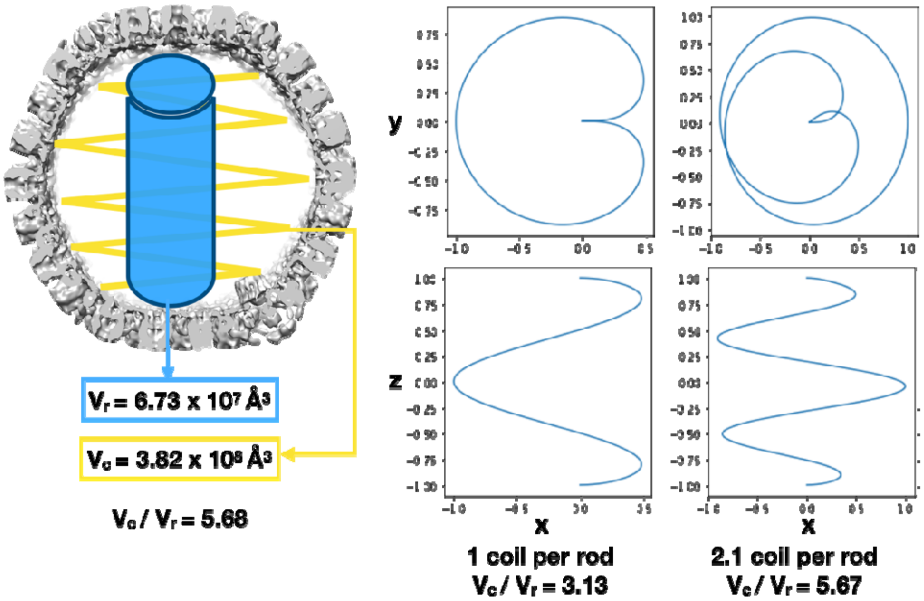


**Fig.S3.** Calculation of the length ratio between dsDNA in the coil versus the rod fold in HSV. The number of coils per rod is determined so that the volume ratio of DNA within the rod and coil matches the observation from HSV particles.

bioRxiv preprint doi: https://doi.org/10.1101/2023.12.15.571939; this version posted December 16, 2023. The copyright holder for this preprint (which was not certified by peer review) is the author/funder, who has granted bioRxiv a license to display the preprint in perpetuity. It is made available under aCC-BY 4.0 International license.

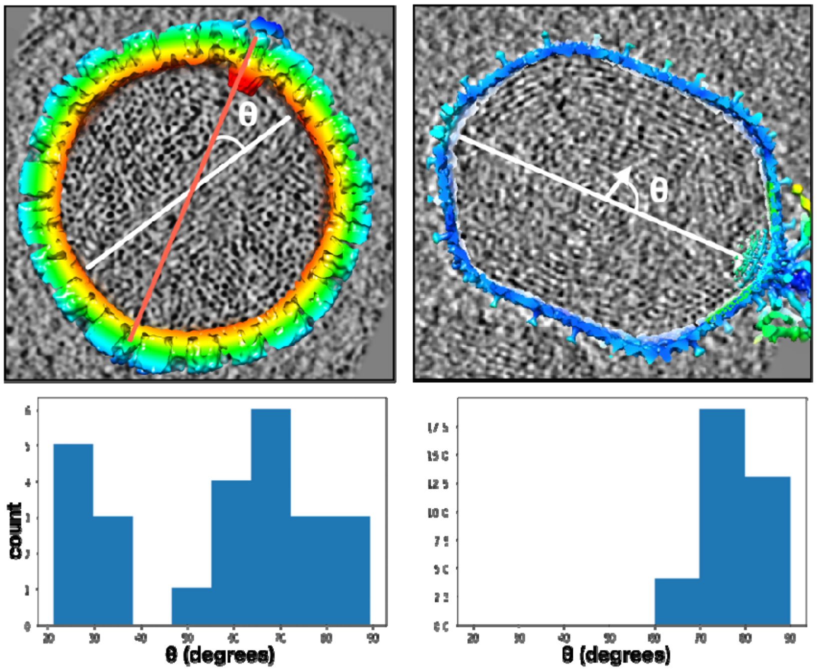


**Fig.S4.** Illustration of the angle between central DNA rod and portal axis in HSV-1 (left) and the T4 bacteriophage (right), and the distribution of the angle among the particles.

bioRxiv preprint doi: https://doi.org/10.1101/2023.12.15.571939; this version posted December 16, 2023. The copyright holder for this preprint (which was not certified by peer review) is the author/funder, who has granted bioRxiv a license to display the preprint in perpetuity. It is made available under aCC-BY 4.0 International license.

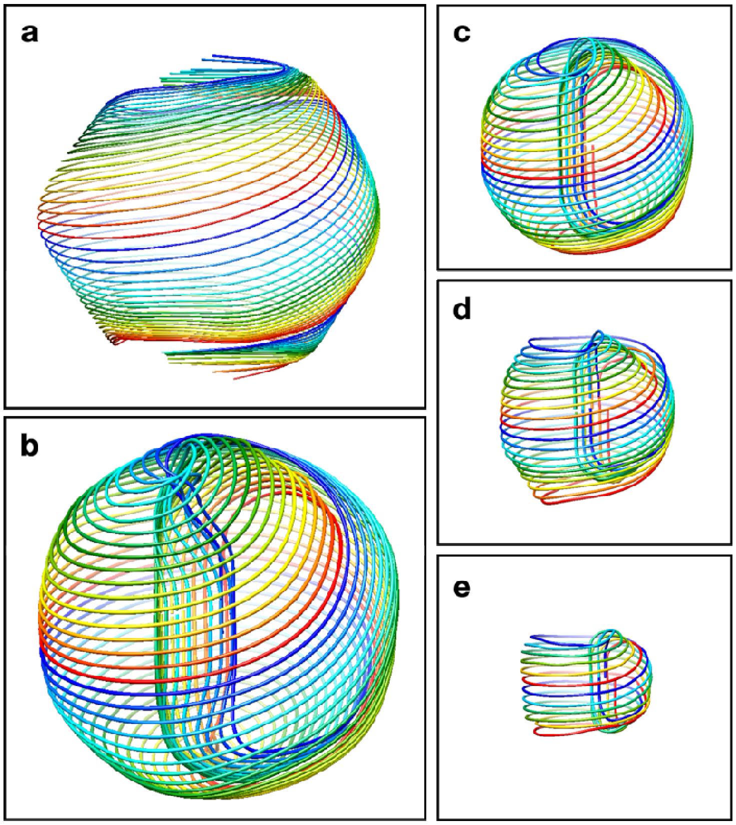


**Fig.S5.** Generation of dsDNA packaging model for HSV-1. (a) Initial path of the DNA strands of the outermost coil layer fit to the shape of the capsid. (b) Final path of the outermost coil layer and corresponding strands in the central rod. (c-e). Final paths of the 3,5,7th coil layers and corresponding strands in the central rod.

bioRxiv preprint doi: https://doi.org/10.1101/2023.12.15.571939; this version posted December 16, 2023. The copyright holder for this preprint (which was not certified by peer review) is the author/funder, who has granted bioRxiv a license to display the preprint in perpetuity. It is made available under aCC-BY 4.0 International license.

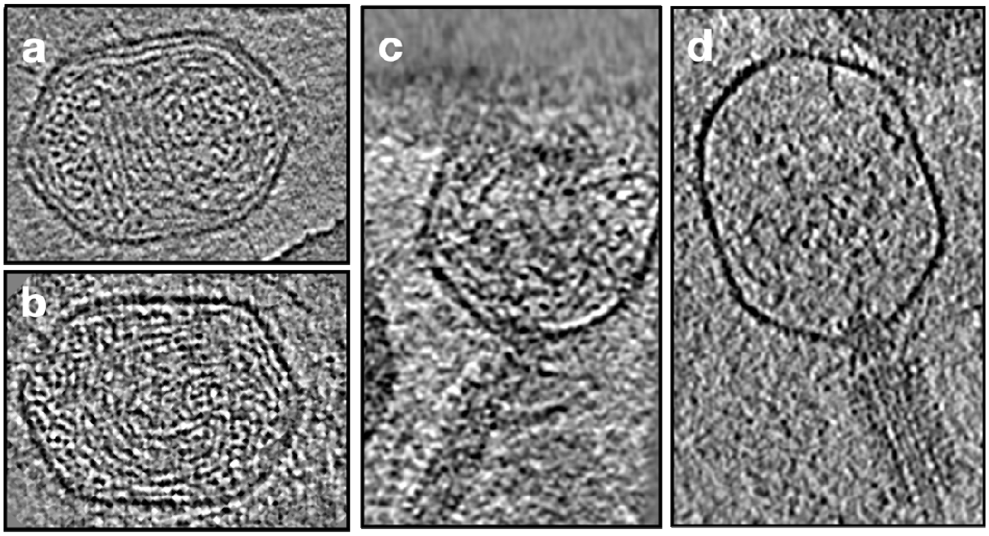


**Fig.S6.** Comparison of T4 genome packaging/releasing intermediates. (a-b) Slice view of T4 particles during the DNA packaging process. The genome density inside the capsid is lower than that of the mature bacteriophage, but the organization is ordered. (c-d) Slice view of T4 particles during the DNA releasing process, showing the disordered DNA inside.

bioRxiv preprint doi: https://doi.org/10.1101/2023.12.15.571939; this version posted December 16, 2023. The copyright holder for this preprint (which was not certified by peer review) is the author/funder, who has granted bioRxiv a license to display the preprint in perpetuity. It is made available under aCC-BY 4.0 International license.

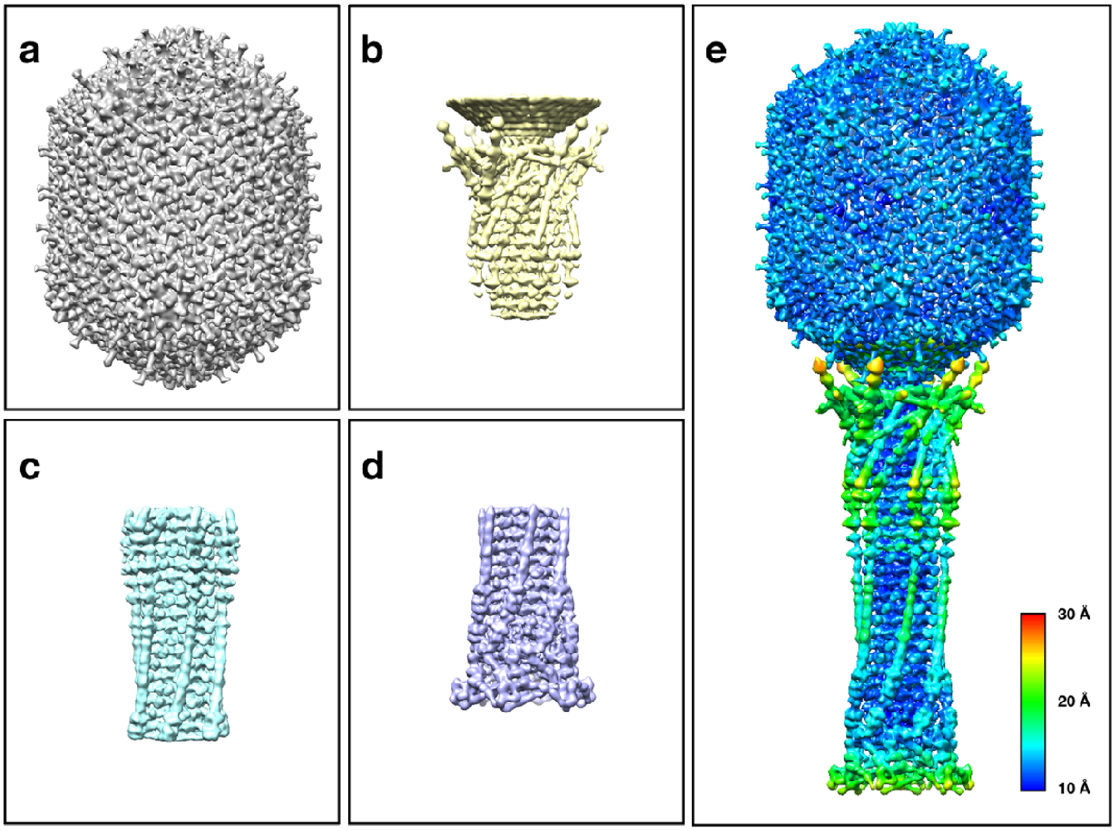


**Fig.S7.** Structure of T4 bacteriophage. (a-d) Subtomogram averages of virus capsid and three segments of the virus tail. (e) Merged structure of the entire bacteriophage, colored by local resolution.

bioRxiv preprint doi: https://doi.org/10.1101/2023.12.15.571939; this version posted December 16, 2023. The copyright holder for this preprint (which was not certified by peer review) is the author/funder, who has granted bioRxiv a license to display the preprint in perpetuity. It is made available under aCC-BY 4.0 International license.

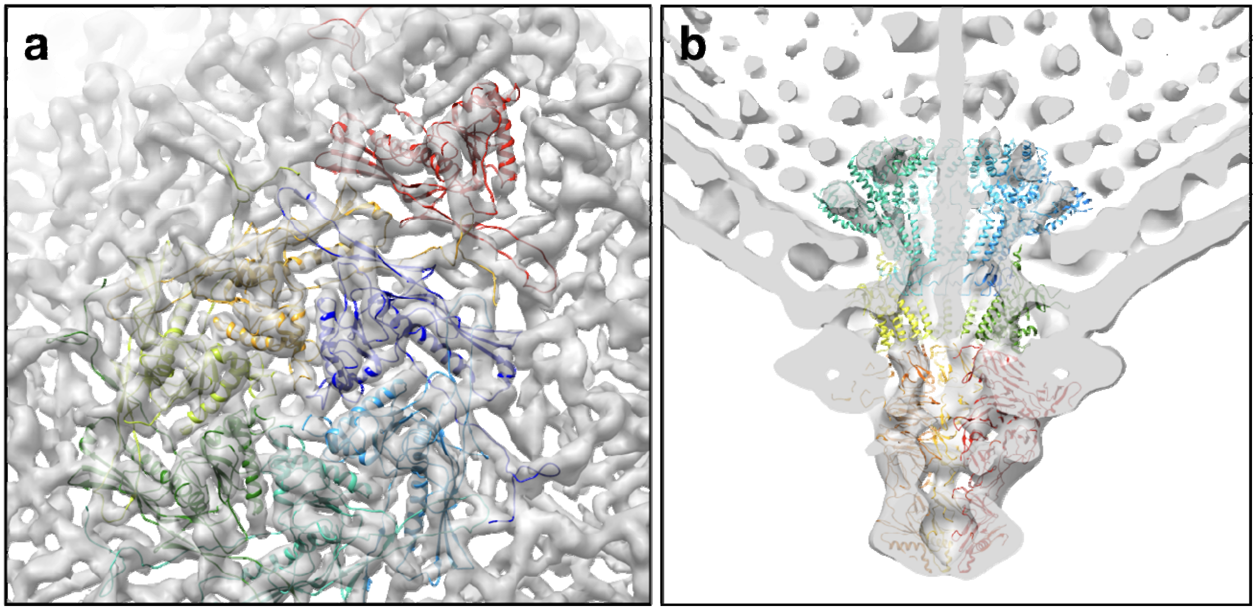


**Fig.S8.** Structure of T7 bacteriophage capsid. (a) Capsid structure determined with icosahedral symmetry with corresponding high resolution model (PDB: 3j7x) fitted into the density. (b) Capsid structure determined without symmetry, showing the portal complex. The corresponding high resolution model (PDB: 6r21) fitted into the density.

bioRxiv preprint doi: https://doi.org/10.1101/2023.12.15.571939; this version posted December 16, 2023. The copyright holder for this preprint (which was not certified by peer review) is the author/funder, who has granted bioRxiv a license to display the preprint in perpetuity. It is made available under aCC-BY 4.0 International license.

Supplementary video 1 - Tomogram slice view of HSV-1
Supplementary video 2 - Slice view of polished HSV-1 particle #1
Supplementary video 3 - Slice view of polished HSV-1 particle #2
Supplementary video 4 - Tomogram slice view of T4 bacteriophage
Supplementary video 5 - Slice view of polished mature T4 particle #1
Supplementary video 6 - Slice view of polished mature T4 particle #2
Supplementary video 7 - Slice view of polished intermediate T4 particle #1
Supplementary video 8 - Slice view of polished intermediate T4 particle #2
Supplementary video 9 - Slice view of polished T7 particle #1
Supplementary video 10 - Slice view of polished T7 particle #2
Supplementary video 11 - 3D view of the DNA packaging model in HSV-1
Supplementary video 12 - 3D view of the morphing from a DNA toroid to the rod-and-coil fold
